# Chronic Kidney Disease Stage Is a Modulator on the Association between High-Sensitivity C-Reactive Protein and Coronary Vasospastic Angina

**DOI:** 10.1155/2014/852507

**Published:** 2014-03-05

**Authors:** Heng-Jung Hsu, Chiung-Hui Yen, Kuang-Hung Hsu, I-Wen Wu, Chin-Chan Lee, Chiao-Yin Sun, Chia-Chi Chou, Chun-Yu Chen, Shih-Ying Yang, Chi-Jen Tsai, Mai-Szu Wu, Ming-Jui Hung

**Affiliations:** ^1^Department of Nephrology, Chang Gung Memorial Hospital, Keelung 20401, Taiwan; ^2^The Graduate Institute of Clinical Medical Sciences, Chang Gung University Medical College, Taoyuan 33302, Taiwan; ^3^Department of Medicine, Chang Gung University, Taoyuan 33302, Taiwan; ^4^Department of Pediatrics, Taipei Medical University Hospital, Taipei 11031, Taiwan; ^5^Laboratory of Epidemiology, Department of Health Care Management, Chang Gung University, Taoyuan 33302, Taiwan; ^6^Department of Cardiology, Chang Gung Memorial Hospital, Keelung 20401, Taiwan

## Abstract

The prevalence of coronary vasospasm and also the factors associated with coronary vasospasm in CKD is still unclear. In this cross-sectional study of 859 consecutive CKD patients with angina pectoris received coronary catheterization, we evaluated the factors associated with coronary vasospasm. Patients with vasospasm were older and had higher peripheral blood white cell counts, higher peripheral blood monocyte cell counts, higher haemoglobin levels, higher hs-CRP levels, and lower levels of serum creatinine than patients without vasospasm. The results of multivariate logistic regression analysis revealed that peripheral blood monocyte count and hs-CRP level were independently associated with coronary vasospasm in patients with stage 1 CKD. Only peripheral blood monocyte count but not hs-CRP was independently associated with coronary vasospasm in patients with stages 2 and 3 of CKD. In conclusion, peripheral blood monocyte count is independently associated with coronary vasospasm in patients with stage 1–3 CKD, whereas hs-CRP is only independently associated with coronary vasospasm in patients with stage 1 CKD.

## 1. Introduction

Chronic kidney disease (CKD) is an independent risk factor for cardiovascular disease [1–3]. Besides, cardiovascular disease-related death is the most common cause of death in CKD patients [4–6]. Diabetes, obesity, and hypertension as well as renal dysfunction, oxidative stress, low-grade inflammation, and dyslipidemia are common pathophysiological mechanisms that play a role in the association between renal failure and cardiovascular disease [3].

Coronary artery spasm plays an important role in the pathogenesis of angina, acute myocardial infarction, arrhythmia, and sudden death [7–10]. The precise mechanism of coronary spasm, however, is not fully understood but seems to be associated with inflammatory disease [11]. Coronary vasospastic angina (VSA) in general population is characterized by the presence of elevated levels of C-reactive protein [12, 13] and also peripheral monocyte counts [14].

CKD is a hyperinflammatory disease characterized by an irreversible deterioration of renal function that gradually progresses to end-stage renal disease. Dysfunction of the immune system induced by the uremic milieu is considered to be the primary cause of hyperinflammation in patients with CKD [15]. Hypercytokinemia is a typical feature of uremia. Accumulation of proinflammatory cytokines as a consequence of decreased renal elimination as well as oxidative stress, volume overload, and comorbidities contributed to hypercytokinemia in patients with CKD. Furthermore, it has been shown that high-sensitivity C-reactive protein (hs-CRP) is elevated in patients with CKD [16].

It is still unknown whether CKD patients with hyperinflammation are high risk group for coronary vasospasm or not. In early CKD patients, Koga et al. found that lower levels of eGFR were significantly and independently associated with high prevalence of coronary artery vasospasm. However, little studies have focused on the factors that might be associated with coronary artery spasm in patients with CKD. The study was to evaluate the prevalence of coronary vasospasm in early and moderate CKD stage patients and also the factors associated with coronary vasospasm in CKD patients.

## 2. Materials and Methods

### 2.1. Study Design

**Figure 1:**
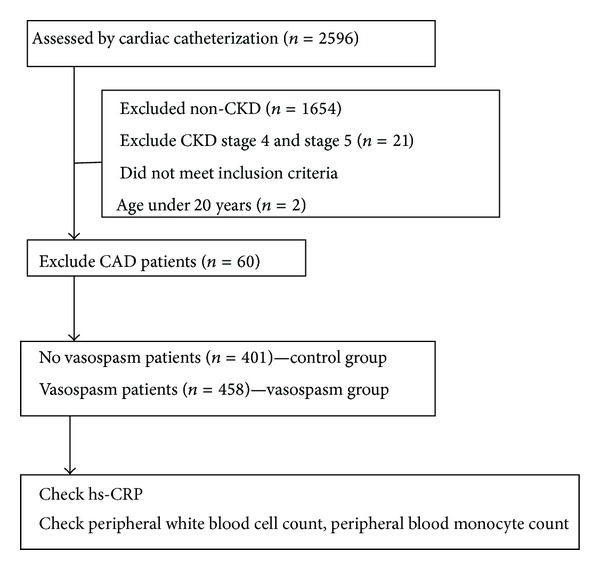
Flow chart indicating patient enrollment and study design.

All patients with angina pectoris with age above 20 years old in this study underwent cardiac catheterization for suspected ischemic heart disease during the period January 1999 to April 2007. We excluded non-CKD patients due to study purpose focusing on CKD. Besides, those patients with advanced CKD stage (CKD stages 4 and 5) were also excluded for preventing contrast nephropathy. We excluded patients with significant CAD in the cardiac catheterization exam (Figure 1). We compared the data from the vasospasm group which was defined as those patients without hemodynamically significant CAD and with evidence of coronary vasospasm on intracoronary ergonovine provocation testing and from control group which was defined as those patients without hemodynamically significant CAD and without evidence of coronary vasospasm on intracoronary ergonovine provocation testing. All eligible patients were carefully interviewed to evaluate the risk factors for cardiovascular disease, including cigarette smoking, diabetes mellitus, hypertension, and total cholesterol levels. Furthermore, patients were stratified by CKD stage using the Modification of Diet in Renal Disease (MDRD) study equation and the NKF KDOQI classification system [17]. We then evaluated the factors that are associated with coronary artery spasm in patients with different stages of CKD.

### 2.2. Study Population

During the period January 1999 to April 2007, 2596 patients who underwent diagnostic coronary angiography for suspected ischemic heart disease and those patients who showed no evidence of hemodynamically significant CAD were subjected to intracoronary methylergonovine testing. Inclusion criteria for coronary VSA included the following: (1) typical angina at rest associated with ST-segment deviation on electrocardiogram and relief provided by sublingual administration of nitroglycerin, (2) no coronary angiographic evidence of hemodynamically significant CAD after intracoronary nitroglycerin administration, [18] and a positive intracoronary methylergonovine provocation test result [18]. Those patients with atypical angina, no evidence of hemodynamically significant CAD, and negative intracoronary methylergonovine provocation test results were defined as control group. Patients with the following conditions were excluded from participation in the study: history of coronary angioplasty or coronary artery bypass graft surgery; evidence of hemodynamically significant CAD with myocardial infarction within the previous 6 months; presence of inflammatory manifestations that are likely associated with noncardiac diseases (e.g., infections and autoimmune disorders); and evidence of liver disease, autoimmune disease, or malignant cancer. This study was approved by the Institutional Review Board of the Chang Gung Memorial Hospital and written informed consent was obtained from all patients before commencement of the study.

### 2.3. Clinical Data

Patients were assessed for the presence of cardiac risk factors, including body mass index (BMI), cigarette smoking, diabetes mellitus, and arterial hypertension. Current smoking status was defined in patients with at least a 1-pack year history of smoking and in patients who had smoked a cigarette within 3 weeks prior to cardiac catheterization. Diabetes mellitus was diagnosed in patients who were using dietary or medical therapy to control blood glucose levels and hypertension was diagnosed in patients who were receiving appropriate medical therapy for hypertension and in patients with a blood pressure of >140/90 mm Hg. Systolic blood pressure and diastolic blood pressure were measured after 15-minute rest before coronary angiography. In addition, left ventricular ejection fraction (LVEF) during coronary angiography was used to assess systolic cardiac function.

### 2.4. Laboratory Analysis

Blood specimens for measurement of hs-CRP were collected in citrate-treated tubes immediately before coronary angiography after overnight fasting and then centrifuged for ≥15 minutes. The plasma component was frozen and transported to the core laboratory on dry ice, where samples were stored at −70°C until subsequent measurements. Serum hs-CRP was measured in duplicate by an enzyme-linked immunosorbent assay of purified protein and polyclonal anti-CRP antibodies (IMMULITE hs-CRP; Diagnostic Products Corp., Los Angeles, California). The lower limit of this assay was 0.10 mg/L and the coefficients of variation were ≤5% at 0.20 mg/L of CRP. We also obtained complete laboratory profiles for patients, including levels of blood creatinine, haemoglobin, peripheral white blood cell count, peripheral blood monocyte count, and total cholesterol. Blood creatinine levels were assessed by spectrophotometric analysis using a modified kinetic Jaffe reaction.

### 2.5. CKD Staging

CKD was diagnosed in patients with persistent proteinuria or an estimated glomerular filtration rate (eGFR) of <90 mL/min per 1.73 m^2^, as determined by the MDRD in two separate measurements within 3 months [17]. We used the NKF KDOQI classification system to classify patients as having stage 1, stage 2, or stage 3 CKD.

### 2.6. Coronary Angiography and Intracoronary Methylergonovine Testing

Nitrates and calcium antagonists were withdrawn for ≥24 hours before coronary angiography. The LVEF was calculated using Simpson's method. Quantitative coronary angiography with an edge-detection algorithm was performed using Judkin's techniques and a Philips digital angiography system (Philips Integris BH 3000; Philips, Bast, The Netherlands). Selective left and right coronary angiography were performed in multiple axial and hemiaxial projections. An independent cardiologist interpreted all coronary angiograms. Hemodynamically significant coronary stenosis was defined as ≥50% diameter reduction in lumen caliber after administration of intracoronary nitroglycerin (50–200 *μ*g). Intracoronary methylergonovine (Methergin; Novartis, Basel, Switzerland) provocation testing was performed in succession if no hemodynamically significant coronary stenosis was demonstrated. Methylergonovine was administered stepwise (1, 5, 10, 30 *μ*g) first into the right coronary artery and then into the left coronary artery. Provocation testing for coronary VSA was considered positive when there was a >70% reduction in luminal diameter with evidence of angina and/or ST depression or elevation changes. In patients in whom coronary VSA was diagnosed, provocation testing was stopped with intracoronary nitroglycerin 50–200 *μ*g (Millisrol; G. Pohl-Boskamp, Hohenlockstedt, Germany). Reversal of coronary artery diameter confirmed the diagnosis of coronary VSA. Spontaneous coronary VSA was defined as an increase in luminal diameter of >70% after intracoronary nitroglycerin administration (50–200 *μ*g) [19].

### 2.7. Statistical Analysis

*t*-tests were used to detect between-group differences in continuous variables and one-way ANOVA was used to compare continuous variables between three groups. The chi-square test was used for categorical comparisons of data. Univariate logistic regression analyses were used to identify risk factors and to estimate odds ratios (OR) and 95% confidence intervals and multivariate logistic regression analyses were used to identify independent risk factors associated with coronary vasospasm. We used Mann Whitney test to compare hs-CRP levels between groups due to hs-CRP levels were not normally distributed. A *P* value < 0.05 was considered to indicate statistical significance; all tests were two tailed. All statistical tests were performed with the statistical package SPSS for Windows (Version 11.0, SPSS Inc., Chicago, Illinois).

## 3. Results

### 3.1. Percentage of Patients with Coronary Vasospasm and Normal Control in Different Stages of CKD

There were 859 consecutive patients included in the final analysis. Among these patients, 401 patients had no hemodynamically significant CAD and no evidence of coronary vasospasm (control group) and 458 patients had coronary vasospastic angina pectoris without hemodynamically significant CAD (vasospasm group) (Table 1). Coronary vasospasm was diagnosed in 49.8% of stage 1 CKD patients, in 49.8% of stage 2 CKD patients, and in 50% of stage 3 CKD patients (Table 1). Normal control group was diagnosed in 44.1% of stage 1 CKD patients, in 44.4% of stage 2 CKD patients, and in 43.6% of stage 3 CKD patients. There were no significant differences in the number of patients with coronary vasospasm and control group among stages 1, 2, or 3 disease (*P* = 0.998).

### 3.2. Inflammatory Marker Levels in Different CKD Stages

We compared the inflammatory markers including hs-CRP and peripheral blood monocyte counts in different CKD stages. It was shown that peripheral blood monocyte counts were similar among patients with different stages of disease (Table 2); however, the levels of hs-CRP were significantly higher among patients with stage 3 CKD than among patients with stage 1 or stage 2 disease (*P* < 0.001).

### 3.3. Characteristics of the Study Population

When we compared the data from patients in the vasospasm group with data from the control group, we found that patients with vasospasm were older (59.5 ± 11.6 versus 56.7 ± 12.6 years, *P* = 0.001), were predominantly male (70% versus 50%, *P* < 0.001), and had a higher rate of cigarette smoking (45% versus 26%, *P* < 0.001) (Table 3). Patients with vasospasm also had significantly higher peripheral white blood cell counts (7.52 ± 2.41 versus 6.57 ± 1.78/1000 mm^3^, *P* < 0.001) and peripheral monocyte blood cell counts (423 ± 364 versus 258 ± 217 mm^3^, *P* < 0.001), higher haemoglobin levels and hs-CRP levels (5.4 ± 7.7 versus 2.8 ± 3.4 mg/L, *P* < 0.001), and lower levels of serum creatinine. There were no significant differences in BMI, the presence of diabetes mellitus (DM), arterial hypertension, eGFR, levels of serum cholesterol, LVEF, or systolic or diastolic blood pressure between patients with vasospasm and control group.

### 3.4. Univariate Analyses of Variables Associated with Coronary Vasospasm in Patients with Different CKD Stages

A total of 8 variables were significantly associated with coronary vasospasm, namely, age, male gender, cigarette smoking, peripheral white blood cell count, peripheral blood monocyte count, haemoglobin, creatinine, and hs-CRP (Table 4). We then stratified patients into CKD stage 1, stage 2, and stage 3 by NKF KDOQI classification. We used above variables to evaluate the factors associated with coronary vasospasm. However, we used eGFR to replace serum creatinine due to that eGFR may be more suitable to characterize renal function. We found that coronary vasospasm in patients with stage 1 CKD was significantly associated with older age, male gender, cigarette smoking, higher peripheral white blood cell counts, higher peripheral monocyte blood cell counts, higher haemoglobin levels, lower eGFR levels, and higher hs-CRP levels. Coronary vasospasm in patients with stage 2 CKD was significantly associated with older age, male gender, cigarette smoking, higher peripheral white blood cell counts, higher peripheral monocyte blood cell counts, and higher hs-CRP levels; however, haemoglobin levels and eGFR levels were not associated with coronary vasospasm in patients with stage 2 disease. Furthermore, coronary vasospasm in patients with stage 3 CKD was significantly associated with male gender, higher peripheral white blood cell counts, and higher peripheral monocyte blood cell counts but was not associated with age, cigarette smoking, haemoglobin levels, eGFR levels, or levels of hsCRP.

### 3.5. Multivariate Analysis of Variables Associated with Coronary Vasospasm in Patients with Different Stages of CKD

Results of the multivariate analysis showed that peripheral blood monocyte count (odds ratio: 1.005, 95% CI: 1.003–1.008, *P* < 0.001) and hs-CRP (odds ratio: 1.114, 95% CI: 1.013–1.224, *P* = 0.026) were independently associated with coronary vasospasm in patients with stage 1 CKD and that peripheral blood monocyte count but not hs-CRP was independently associated with coronary vasospasm in patients with stage 2 CKD (odds ratio: 1.005, 95% CI: 1.002–1.007, *P* < 0.001) and in patients with stage 3 disease (odds ratio: 1.006, 95% CI: 1.006–1.012, *P* = 0.026) (Table 5).

**Figure 2:**
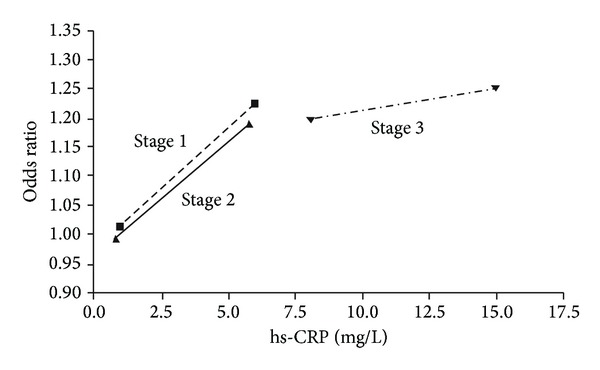
Multivariate-adjusted odds ratios for high-sensitivity C-reactive protein (hs-CRP) in different CKD stages. Hs-CRP was expressed in different CKD stages with value ranges from mean ± standard deviation.

Figure 2 revealed the association between multivariate-adjusted odds ratio of coronary vasospasm and hs-CRP. The correlation of risk of coronary vasospasm and hs-CRP was different in CKD stage 1, stage 2, and stage 3. In CKD stage 1 and stage 2 patients, the serum hs-CRP levels were around 1.0–6.5 mg/L and higher association of hs-CRP with coronary vasospasm was noted (although *P* = 0.067 in CKD stage 2) than stage 3 CKD patients. In CKD stage 3 patients, the serum hs-CRP levels were around 7.5–15.0 mg/L and the association of hs-CRP and coronary vasospasm was not significantly obvious.

## 4. Discussion

The percentage of coronary vasospasm in CKD patients is around 50%. In our previous study in non-CKD patients, the percentage of coronary vasospasm is around 30% [14]. Koga et al. also showed similar percentage of coronary vasospasm in their study in Japan with around 30% in patients with eGFR >75 mL/min/1.73 m^2^ and around 50% in patients with eGFR <75 mL/min/1.73 m^2^ and >64 mL/min/1.73 m^2^ [20]. Those results all suggested that those CKD patients are high risk patients for coronary vasospasm.

In CKD patents with different stages, we found that the percentage of coronary vasospasm was around 50% in CKD stage 1, stage 2, and stage 3 patients without difference between stages. Koga et al. found that low glomerular filtration rate is associated with high prevalence of vasospastic angina. The different result may be due to different CKD stage of patients' enrollment between our study and Koga et al. study. Koga et al. enrolled early stage CKD (stages 1 and 2) whereas our study enrolled CKD stage 1, stage 2, and also stage 3 patients. The different study population may let the result be diverse. Besides, racial different may also be one explanation for the dissimilar results.

Coronary vasospasm is a multifactorial condition. In this study, we tried to determine the factors that are associated with coronary vasospasm in patients with early stage CKD (stages 1 and 2) and in patients with moderate CKD (stage 3). We found that peripheral blood monocyte count was independently associated with coronary vasospasm in patients with stage 1–3 CKD and that hs-CRP level was independently associated with coronary vasospasm only in patients with stage 1 CKD, although serum levels of hs-CRP were similar between patients with stage 1 CKD and patients with stage 2 disease and were significantly higher in patients with stage 3 disease. Coronary vasospasm is considered to an inflammatory disease with endothelium injury. During injury of endothelium injury, endothelium may secret chemokines which trigger monocyte migration and adhesion to the injured endothelium to initiating atherosclerotic lesion and progression [21–23]. The study found that the association between inflammatory marker-hs-CRP was only significant in CKD stage 1 patients but loss significance in CKD stage 2 and CKD stage 3 patients though there was a trend in CKD stage 2 patients. The effect of uremic toxins may partly explain these findings. Accumulation of uremic toxins can have detrimental effects on health and lead to uremic syndrome, cardiovascular disease, inflammation, and increased mortality [24, 25]. Alterations in the immune system in CKD by uremia are associated with a state of immune dysfunction characterized by immune-depression that contributes to the high prevalence of infections among these patients, as well as by immune-activation resulting in inflammation that may contribute to cardiovascular disease [15]. Serum levels of protein-bound uremic toxins such as P-Cresol sulfate [PCS] and indoxyl sulfate [IS] have been shown to be elevated in patients with poor renal function [26]. Furthermore, several studies have shown that PCS and IS are associated with endothelial dysfunction, vascular disease, and immune dysregulation in patients with renal disease [27]. Uremic toxins accumulation maybe interfere with the inflammatory process of coronary vasospasm during endothelium injury. As renal function downhill from CKD stage 1 to stage 3, more uremic toxins accumulate and then possible the association between hs-CRP and coronary vasospasm lost its significance. Furthermore, vascular calcification in more advanced CKD stage may be another explanation for higher hs-CRP levels and no significant association between hs-CRP and coronary vasospasm in CKD stage 3 patients. Vascular calcification is associated with less coronary vasospasm [28]. Vascular calcification, which highly related to calcium/phosphate imbalance, is most common in CKD stages 3, 4, and 5 [29]. Therefore, the effect of hs-CRP on coronary vasospasm may be offset by vascular calcification in patients with moderately reduced kidney function who present with high levels of hs-CRP.

We found that coronary vasospasm was associated with lower eGFR in patients with stage 1 CKD but not in patients with stage 2 or stage 3 disease. Koga et al. also found that lower eGFR was a potential risk factor for vasospastic angina in patients with early stage CKD patients [20]. Combining our result and Koga et al. study, we can realize the complexity of association between renal function and vasospastic angina in CKD patients. It seems that lower eGFR was associated with coronary vasospasm in early CKD patients. However, when renal function keeps downhill to CKD stage 3, the association between eGFR and coronary vasospasm was not prominent.

This study found that peripheral monocyte count is significantly associated with coronary vasospasm in CKD stage 1, stage 2, and stage 3. This result was compatible with our previous study which found that hs-CRP and peripheral monocyte count was independently significantly associated with coronary vasospasm [11]. But the association between coronary vasospasm and inflammatory makers such as hs-CRP and peripheral monocyte count was more complicated. In CKD patients, hyperinflammation and vascular calcification are common. Besides, uremic toxins accumulation is also noted in CKD patients. This complicated situation in CKD patients lets lots of study exclude CKD patients. This study is the first study which is focusing on vascular spasm in CKD patients with association with hs-CRP. Besides, our patients' number is large. In this study, the associations between coronary vasospasm and inflammatory markers such as hs-CRP and peripheral monocyte count were significantly associated coronary vasospasm in CKD stage 1 patients. But the association between coronary vasospasm and hs-CRP lost significance in CKD stage 2 and stage 3 patients. The study further indicated that CKD patients are a specific population that is very different from the general population. The clinical data or principle cannot be simply extra-plotted from general population to CKD patients.

## 5. Conclusion

Peripheral blood monocyte count is independently associated with the development of coronary vasospasm in patients with CKD stages 1–3 and hs-CRP is independently associated with coronary vasospasm in patients with CKD stage 1. Furthermore, coronary vasospasm is significantly associated with lower eGFR in patients with stage 1 CKD but not in patients with stage 2 or stage 3 diseases.

## Figures and Tables

**Table 1:** Percentage of normal patients and patients with CAD and vasospasm according to chronic kidney disease stage.

	CKD stage 1	CKD stage 2	CKD stage 3	*P* value
	*N* = 245	*N* = 518	*N* = 156	
Coronary artery disease	15 (6.1%)	35 (6.8%)	10 (6.4%)	0.998
Coronary vasospasm	122 (49.8%)	258 (49.8%)	78 (50%)	
Normal	108 (44.1%)	225 (43.4%)	68 (43.6%)	

Statistical significance based on chi-square test. CAD: coronary artery disease; CKD: chronic kidney disease.

**Table 2:** The laboratory data about peripheral monocyte blood cell count and hs-CRP according to CKD stage.

	CKD stage 1	CKD stage 2	CKD stage 3	*P* value^#^
	*N* = 245	*N* = 518	*N* = 156	
Peripheral monocyte blood cell count, /mm^3^	336 ± 260	356 ± 349	397 ± 271	0.314
hs-CRP, mg/L	4.30 ± 5.98	4.19 ± 5.36	8.15 ± 14.53^†‡^	<0.001

Data are presented as mean ± standard deviation.

^#^Statistical significance based on one-way ANOVA analysis for continuous variables.

^†^ *P* < 0.05 between CKD stage 1 patients and CKD stage 3 patients.

^‡^ *P* < 0.05 between CKD stage 2 patients and CKD stage 3 patients.

Statistical significance of peripheral monocyte blood cell count based on *t*-test for continuous variables.

Statistical significance of hs-CRP based on Mann Whitney test for continuous variables due to hs-CRP levels was not normally distributed.

CKD: chronic kidney disease; hs-CRP: high-sensitive C-reactive protein.

**Table 3:** Baseline characteristics of study patients.

Variable	All patients	Control	Vasospasm	*P* value
	*N* = 859	*N* = 401	*N* = 458	
Age, years	58.0 ± 12.1	56.7 ± 12.6	59.5 ± 11.6	0.001*
Men, *n* (%)	538 (63%)	211 (50%)	327 (70%)	<0.001*
Body mass index, kg/m^2^	26.2 ± 3.9	26.1 ± 4.3	26.2 ± 3.6	0.629
Smoker, *n* (%)	312 (36%)	106 (26%)	206 (45%)	<0.001*
Diabetes mellitus, *n* (%)	189 (22%)	86 (21%)	103 (22%)	0.434
Arterial hypertension, *n* (%)	404 (47%)	177 (44%)	227 (50%)	0.105
Peripheral white blood cell count, /1000 mm^3^	7.13 ± 2.19	6.57 ± 1.78	7.52 ± 2.41	<0.001*
Peripheral monocyte blood cell count, /mm^3^	346 ± 316	258 ± 217	423 ± 364	<0.001*
Haemoglobin, g/dL	13.6 ± 1.6	13.2 ± 1.8	13.7 ± 1.7	<0.001*
Creatinine, mg/dL	1.2 ± 0.3	1.3 ± 1.8	1.1 ± 0.9	0.049*
eGFR, mL/min/1.73 m^2^	77.3 ± 11.5	77.0 ± 25.5	77.8 ± 22.4	0.796
CKD stage				0.995
1, *n*, %	230	108 (27%)	122 (27%)	
2, *n*, %	483	225 (56%)	258 (56%)	
3, *n*, %	146	68 (17%)	78 (17%)	
^&^hs-CRP, mg/L	4.4 ± 6.5	2.8 ± 3.4	5.4 ± 7.7	<0.001*
Cholesterol, mg/dL	204.3 ± 43.3	204.6 ± 45.0	203.2 ± 42.5	0.633
Left ventricular ejection fraction, %	67 ± 10	68 ± 11	67 ± 9	0.757
Systolic blood pressure, mmHg	131 ± 19	131 ± 20	132 ± 19	0.64
Diastolic blood pressure, mmHg	77 ± 12	78 ± 12	77 ± 11	0.295

Data are presented as mean ± standard deviation or number of patients (percentage).

*P < 0.05 between patients who did not have vasospasm and patients who did.

^&^Mann Whitney test was used to compare hs-CRP levels between groups due to that hs-CRP levels were not normally distributed.

Abbreviation: eGFR: estimated glomerular filtration rate; CKD: chronic kidney disease; hs-CRP: high sensitivity-C reactive protein.

Statistical significance based on Student's *t*-test or chi-square test.

**Table 4:** Univariate analysis of variables associated with coronary vasospasm in the study patients.

Variables	All patients			CKD stage 1			CKD stage 2			CKD stage 3		
	*N* = 458/859			*N* = 122/230			*N* = 258/483			*N* = 78/146		
	Odds ratio	95% CI	*P* value	Odds ratio	95% CI	*P* value	Odds ratio	95% CI	*P* value	Odds ratio	95% CI	*P* value
Age, years	1.02	1.008–1.031	0.001	1.041	1.015–1.067	0.002	1.019	1.003–1.035	0.019	1.017	0.947–1.041	0.701
Men versus women	2.429	1.835–3.215	<0.001	2.431	1.778–3.324	<0.001	2.245	1.529–3.297	<0.001	2.668	1.360–5.234	0.004
Smoker (yes versus no)	2.48	1.849–3.326	<0.001	2.655	1.920–3.672	<0.001	2.355	1.595–3.475	<0.001	1.794	0.895–3.598	0.1
Peripheral white blood cell count, /1000 mm^3^	1.244	1.143–1.353	<0.001	1.221	1.112–1.339	<0.001	1.21	1.087–1.347	0.001	1.356	1.098–1.675	0.005
Peripheral monocyte blood cell count, /mm^3^	1.003	1.002–1.003	<0.001	1.003	1.002–1.004	<0.001	1.002	1.001–1.003	<0.001	1.002	1.001–1.004	0.007
Haemoglobin, g/dL	1.152	1.045–1.271	0.004	1.147	1.026–1.283	0.016	1.141	0.998–1.304	0.053	1.207	0.970–1.502	0.091
eGFR, mL/min/1.73 m^2^	0.997	0.991–1.003	0.36	0.984	0.975–0.994	0.001	0.992	0.983–1.001	0.075	0.986	0.966–1.006	0.175
hs-CRP, mg/L	1.133	1.067–1.202	<0.001	1.15	1.070–1.236	<0.001	1.186	1.078–1.304	<0.001	1.09	0.987–1.204	0.089

Statistical significance based on binary logistic regression analysis. CKD: chronic kidney disease; eGFR: estimated glomerular filtration rate; hs-CRP: high-sensitivity C-reactive protein.

**Table 5:** Multivariate analysis of factors associated with coronary vasospasm in the study patients in different CKD stages.

Variables	CKD stage 1			CKD stage 2			CKD stage 3		
	*N* = 122/230			*N* = 258/483			*N* = 78/146		
	Odds ratio	95% CI	*P* value	Odds ratio	95% CI	*P* value	Odds ratio	95% CI	*P* value
Age, years									
Men versus women									
Smoker (yes versus no)									
Peripheral white blood cell count, /1000 mm^3^									
Peripheral monocyte blood cell count, /mm^3^	1.005	1.003–1.008	<0.001	1.005	1.002–1.007	<0.001	1.006	1.001–1.012	0.026
Haemoglobin, g/dL									
eGFR, mL/min/1.73 m^2^									
hs-CRP, mg/L	1.114	1.013–1.224	0.026	1.097	0.994–1.210	0.067			

*Multivariate-adjusted odds ratio adjusted by age, male gender, smoker, peripheral white blood cell count, peripheral monocyte blood cell count, haemoglobin, eGFR, and hs-CRP.

Statistical significance based on binary logistic regression analysis.

CKD: chronic kidney disease; eGFR: estimated glomerular filtration rate; hs-CRP: high-sensitivity C -reactive protein.
